# A Metabolomics Exploration of the Sexual Phase in the Marine Diatom *Pseudo-nitzschia multistriata*

**DOI:** 10.3390/md18060313

**Published:** 2020-06-14

**Authors:** Federica Fiorini, Camilla Borgonuovo, Maria Immacolata Ferrante, Mark Brönstrup

**Affiliations:** 1 Department of Chemical Biology, Helmholtz Centre for Infection Research, Inhoffenstrasse 7, 38124 Braunschweig, Germany; federica.fiorini@helmholtz-hzi.de; 2 Integrative Marine Ecology Department, Stazione Zoologica Anton Dohrn, Villa Comunale, 80121 Naples, Italy; camilla.borgonuovo@szn.it; 3 Center of Biomolecular Drug Research (BMWZ), Leibniz Universität, 30167 Hannover, Germany

**Keywords:** marine natural products, metabolomics, mass spectrometry, diatoms, *Pseudo-nitzschia multistriata*, sexual reproduction

## Abstract

*Pseudo-nitzschia multistriata* is a planktonic marine diatom with a diplontic life cycle comprising a short sexual phase, during which gametes are produced following the encounter of two diploid cells of opposite mating type (MT). Gene expression studies have highlighted the presence of substantial changes occurring at the onset of sexual reproduction. Herein, we have hypothesized that the amount and nature of cellular metabolites varies along the mating process. To capture the metabolome of *Pseudo-nitzschia multistriata* at different harvesting times in an unbiased manner, we undertook an untargeted metabolomics approach based on liquid chromatography–tandem mass spectrometry. Using three different extraction steps, the method revealed pronounced differences in the metabolic profiles between control cells in the vegetative phase (MT+ and MT−) and mixed strains of opposite MTs (cross) undergoing sexual reproduction. Of the 2408 high-quality features obtained, 70 known metabolites could be identified based on in-house libraries and online databases; additional 46 features could be classified by molecular networking of tandem mass spectra. The reduction of phytol detected in the cross can be linked to the general downregulation of photosynthesis during sexual reproduction observed elsewhere. Moreover, the role of highly regulated compounds such as 7-dehydrodesmosterol, whose changes in abundance were the highest in the experiment, oleamide, ectoine, or trigonelline is discussed.

## 1. Introduction

Marine organisms produce a large variety of chemical compounds involved in several important processes like defense, intra- and inter- specific communication, sexual reproduction and feeding. These so-called secondary metabolites therefore contribute to shaping aquatic communities and have a prominent ecological role [1]. The growing interest in compounds deriving from marine organisms, which are relatively little explored and exploited, is also motivated by the potential to discover new bioactive molecules [2]. Among marine organisms, microalgae seem particularly promising, since they can be easily grown on a large scale under indoor or outdoor conditions, and in principle allow sustained production of a given compound of interest using solar light without deplenishing natural resources.

Diatoms are one of the most important groups of marine microalgae. They are ubiquitous in the oceans and often dominate phytoplankton assemblages, and their contribution to global primary production, ranging around 20%, is considered equivalent to that of terrestrial rain forests [3]. The interest in this group of microalgae has gradually led to the accumulation of a great deal of genomic, physiological and biochemical information, and to the development of tools for genetic manipulation [4,5]. In seminal studies on their chemical ecology, it was found that broken diatom cells release polyunsaturated aldehydes, which can be very toxic for the reproduction of their grazers, the copepods [6], or can serve as signals for bloom termination through the induction of cell death [7]. Other important compounds for diatoms are sex pheromones, molecules that allow the species-specific recognition of opposite ‘sexes’ and the synchronization for gametes production. Sex pheromones in diatoms have been described in a benthic species, *Seminavis robusta*, which has two opposite ‘sexes’ called mating types (MT). In this species, a pheromone called SIP+ (sex inducing pheromone +) is produced by MT+ cells to stimulate MT− cells [8], which in turn produce a second pheromone, the small amino acid diproline, which strongly attracts MT+ cells to promote pairing [9]. No other data on diatom pheromones are available so far.

In order to investigate the chemical diversity of microalgae, and to identify chemical regulators within communities in an unbiased manner, untargeted metabolomics has evolved as a powerful technology [10,11,12]. Recent efforts have been devoted to enlarge the collection of data on microalgae secondary metabolites [13]. The diversity of diatom lipids was assessed in 13 diatom cultures grown under nitrogen deplete and nitrogen replete conditions, revealing that the different growth conditions affected metabolite accumulation, but not the species-specific metabolomic profile [14]. In another study, RNA-sequencing and metabolomics were used to explore the response of diatoms to the presence of grazers, comparing cultures of *Skeletonema marinoi* in the presence of copepods with cultures without grazers. A total of 242 regulated metabolites were identified and among these, a set of compounds downregulated in the treatment with the grazers were annotated as oxylipins, in line with expression data that coherently showed downregulation of enzymes involved in oxylipins production [15].

For the present study, we have selected the planktonic marine diatom *Pseudo-nitzschia multistriata*. The cosmopolitan genus *Pseudo*-*nitzschia* is known to produce the neurotoxin domoic acid, a small compound that competes with glutamate and affects neuronal cell functionality. This leads to neurological symptoms in mammals and birds, which consume contaminated seafood during the toxic algal blooms [16]. *P. multistriata* has a very well described life cycle and has served as a model for genetic studies. Similarly to *S. robusta* and to most of the diatoms, *P. multistriata* is diploid and the only haploid stages are the gametes, produced when two cells of opposite MT encounter each other [17]. Its genome has been fully sequenced, and a number of gene expression data are available [18,19,20]. The species can be transformed [21] and the mechanisms of sex determination as well as the first mating type-determining gene for diatoms have been identified [22]. Gene expression studies have highlighted that profound changes occur at the onset of sexual reproduction: the entire cell population experiences a growth arrest accompanied by the downregulation of cyclins and nutrient transporters, and the upregulation of meiotic genes and of signaling proteins [20]. The MT− experiences more changes than the MT+ in the early phases of sexual reproduction, perhaps due to a temporal misalignment of the process of meiosis between the two MTs.

We hypothesized that during sexual reproduction substantial changes occur also in the amount and nature of the metabolites produced by cells. Therefore, we undertook a metabolomics approach using liquid chromatography coupled to mass spectrometry (LC–MS) to study the differences in the metabolic profiles between control cells in the vegetative phase and mixed strains of opposite MTs undergoing sexual reproduction. The effects of extraction solvents and harvesting times on the ensemble of detected and structurally annotated endo (i.e. intracellular) metabolites were also captured.

## 2. Results

**Figure 1:**
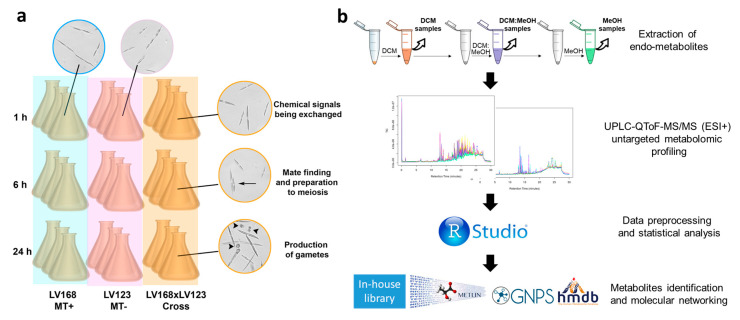
(**a**) Experimental set up used in this study. The mating type + (MT+) strain LV168 and the mating type – (MT−) strain LV123 were grown as monocultures (blue or pink shade) or mixed to induce sexual reproduction (cross, yellow shade). Triplicates were prepared for each sample at each time point, for a total of 27 flasks. Sampling points were 1 h, 6 h, and 24 h after mixing the two monocultures. Images on the top show cells in monocultures, these vegetative samples have the same appearance at the three time points, except that cell density was higher at the last time point because mitotic divisions occurred during the 24 h. Images on the right show cells in the cross at the different time points, at 1 h cells of each MT are signaling their presence, at 6 h paired cells can be seen (arrow), while gametes are visible at 24 h (arrowheads). More details on the timing of this process can be found in [20]. (**b**) Analytical workflow of the untargeted metabolomic study, see also Materials and Methods section. The different steps of the metabolomic analysis pipeline are schematized and include samples preparation via three subsequent extractions of endo-metabolites (dichloromethane, DCM, followed by a combination of dichloromethane:methanol = 1:1, DCM:MeOH, and pure methanol, MeOH), ultra-high-performance liquid chromatography–tandem mass spectrometry in positive ionization mode (UPLC–QToF–MS/MS ESI+) measurement, data analysis, feature detection, statistical evaluation and compound identification.

Briefly, two strains of opposite MT were grown as monocultures or mixed together to induce sexual reproduction. Samples were grown in triplicate and collected 1 h, 6 h, and 24 h after mixing the two monocultures, in order to capture changes occurring when the cells of each MT are signaling their presence, are actively looking for a cell of opposite MT to pair with, or are forming gametes, respectively (Figure 1a). LC–MS-based metabolomics (Figure 1b) was used to detect metabolites of *Pseudo-nitzschia multistriata* produced in two mating types (MT+ and MT−) grown separately or together in the cross (from now on referred to as MTx).

Overall, 81 samples were generated: for each of the three groups, three solvent extraction systems and three time points of harvesting were investigated in three biological replicates.

### 2.1. Effect of Sequential Solvent Extractions on the Endo-Metabolome 

**Figure 2:**
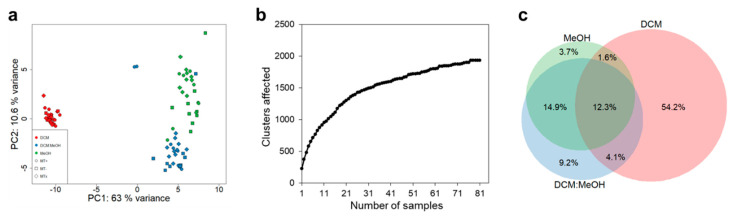
(**a**) Principal component analysis (PCA) obtained from processed data showing relationship between samples treated with three different solvent systems subsequent extractions (DCM in red, DCM:MeOH in green and MeOH in blue). Circles indicate MT+, squares MT− and rhombi MTx. (**b**) Rarefaction curve obtained from Global Natural Products Social Molecular Networking (GNPS, see Materials and Methods section) of MS/MS spectra diversity for the global experiment (all extraction solvents). (**c**) Euler diagram obtained from GNPS (see Materials and Methods section) indicating the overlap between the used solvent systems; percentages of extracted features are shown for each sector.

Extractions were carried out in a sequence of increasing polarity (dichloromethane, DCM; dichloromethane:methanol, DCM:MeOH; methanol, MeOH). Different metabolites were extracted and made accessible by this procedure, as indicated by the principal component analysis of scores (PCA, Figure 2a), which grouped samples mainly by extraction method. The most evident separation in the PCA space occurred between extractions with DCM and those with the other two solvent systems. Such variation clearly highlights the importance of extraction solvents which have a greater effect with respect to cultures characteristics and MT for the overall metabolome. 

The accumulation of new features was visualized as a rarefaction curve (Figure 2b), which uses unique MS/MS spectra as an indicator for hitherto non-represented features added by each additional sample; thus, reflecting the diversity within the investigated dataset. Saturation of the detected chemical space (i.e. curve reaching asymptote) was approached, but not fully reached. This indicates that most of the metabolite diversity is covered with the chosen experimental setup based on three different extraction solvent systems.

A Euler diagram (Figure 2c) confirmed on a feature level what was already observed by PCA on sample-level: the most evident separation is achieved between extractions performed with DCM vs. extractions performed with DCM:MeOH and MeOH. Moreover, DCM extracts captured the majority of metabolites, and yielded the highest number of unique features, followed by DCM:MeOH system and finally by MeOH. 

### 2.2. Identification of Known Metabolites 

Overall, 2408 high-quality features were obtained for the whole experiment across 81 samples, including isotopes and different adducts of the same metabolite (full list available in the Supplementary Materials, file “Processed feature table”).

Known metabolites were first identified using an in-house library of compounds measured under the same conditions. In a second step, the annotation of known metabolites was broadened by searching their exact masses and comparing their MS/MS spectra with online spectral databases to obtain putative metabolite identifications.

**Figure 3:**
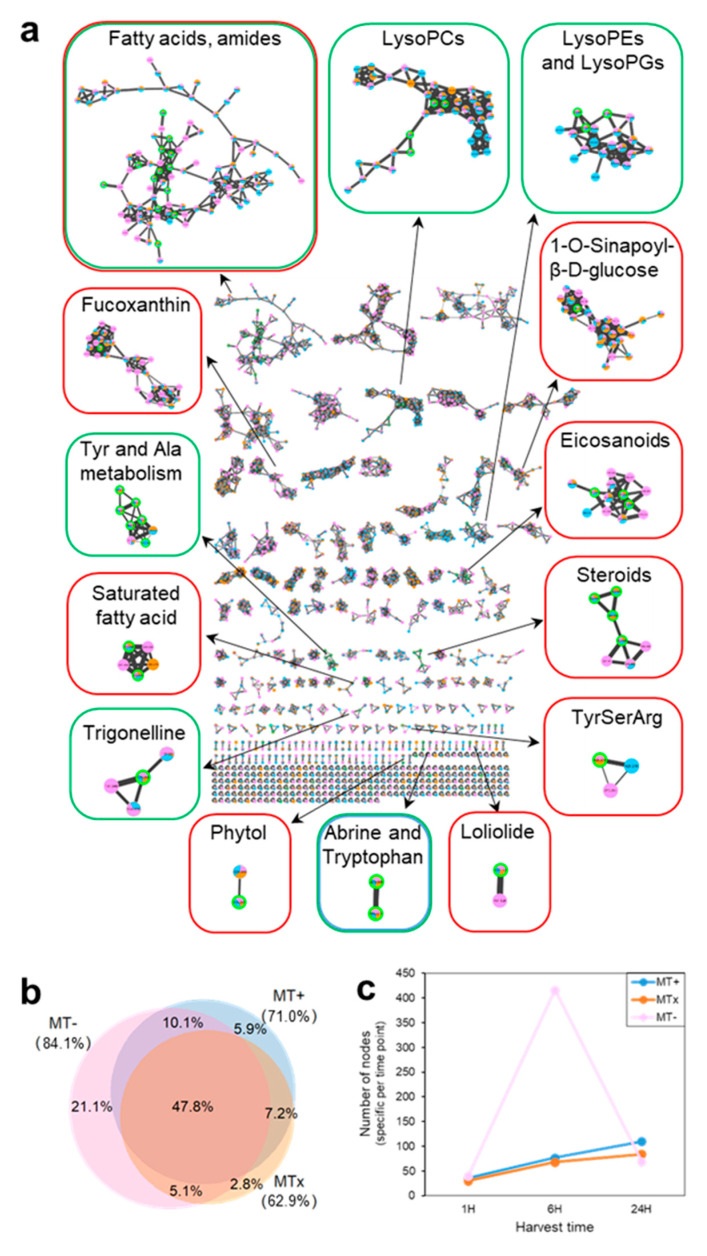
(**a**) Global molecular network of MS/MS spectra from all samples. The color of the nodes indicates the sample type (pink for MT−, light blue for MT+ and orange for MTx). The relative abundance of sample types, as measured by spectral counts, is visualized through pie charts on each node. Identified/annotated nodes are highlighted with a green border; families containing at least one annotation are boxed and color-coded according to the extraction solvent (red for DCM, green for DCM:MeOH, and blue for MeOH). A high resolution version of the MN in (**a**) is added as a supplementary file. (**b**) Euler diagram, based on the global molecular network, indicating the nodes distribution per sample type; percentages are shown for each sector and total percentage for each sample type is reported in parenthesis. (**c**) Number of nodes detected at a specific time point. Nodes are considered for each sample type separately, regardless whether they are present also in other samples; for a given sample type, the number of nodes that is present exclusively at a given time point is plotted.

In order to visually display the global chemical space and structural similarity of the metabolome, and ultimately to further extend the annotation, a comprehensive molecular network (MN) was generated by merging identical MS/MS spectra into single consensus nodes and connecting nodes by an edge when they shared at least six common fragment ions and had a cosine score of 0.7 or more (Figure 3a). The obtained molecular network was composed of 1942 nodes and 199 subfamilies of at least 2 nodes. It is important to underline that the number of nodes of the MN is larger than the number of extracted endo-metabolites, because different adduct ions or different charge states are displayed as individual nodes, even when belonging to the same compound. 

The work of identification and annotation of known metabolites, based on the in-house library, online databases and MS/MS fragmentation similarities resulted in a list of 80 features representing identified or putatively identified compounds from 14 molecular families and single nodes (Table S1). After removal of redundant information, 70 known metabolites were identified. Moreover, 46 additional features were classified as structurally similar to known metabolites based on the MN approach, because they belong to one of the molecular families for which at least one metabolite is identified. Thus, the chemical class is annotated on the basis of the MS/MS fragmentation similarity (Table S2).

### 2.3. Mating Type and Harvesting Time Points

To have an overview of molecular diversity between the two mating types grown in monoculture (MT+ or MT−) and the same strains together undergoing sexual reproduction (MTx), their contribution to the global MN was assessed by comparing the number of nodes detected per sample type, irrespective of the extraction solvent.

According to the Euler diagram depicting the percentage of nodes detected for each sample type (Figure 3b), the overlap of metabolites between sample types is quite high, as 47.8% of the parent ions are shared between MT+, MT−, and MTx. The majority of these shared known nodes correspond to fatty acids or amides, lysophosphatidylcholines and lipophosphoglycans, eicosanoids, steroids, or metabolites from tyrosine (Tyr) and alanine (Ala) metabolic pathways. The toxin domoic acid was also detected in all three samples. The intensity of this metabolite was found to increase over time in MT+ and MT−, while it remained quite constant in the cross sample (Figure S1).

The diagram in Figure 3b also highlights the presence of nodes that are specific to a single MT, indicating metabolites which are produced and detected from only a MT. The MT with the highest molecular diversity is MT−, which displays the highest number of MT specific parent ions (21.1%). The MT− population also accounts for the highest total number of nodes detected within the whole experiment. The lowest number of sample specific nodes is found for MTx (2.8%), followed by MT+ (5.9%).

Samples were harvested at different time points to follow changes occurring at different stages of the process of sexual reproduction (Figure 1a) to capture the kinetics of metabolite expression over time. In the MTx and MT+ population the number of extracted ions grew steadily over time (Figure 3c). The number of nodes generated by MT− was strongly increased at the second time point (6 h), i.e. over 400 nodes. This rise for MT− was also observed by measuring peak intensities of MS1 spectra as a proxy for the abundance of a feature (Figure S2). Notably, the increase of feature abundance in MT− after 6 h and 24 h was mainly caused by species of low molecular weight (<300 Da). This implies that changes occur presumably in the de novo biosynthesis of small metabolites, rather than in the assembly of large biomolecular structures (e.g. lipids, peptides, or oligosaccharides).

### 2.4. Differences between Sample Types in DCM Extracts

In order to investigate the key drivers of the diversity between sample types, we focused on features extracted only with DCM, since it accounts for the majority of extracted metabolites. Variations in metabolite accumulation were investigated by pairwise comparisons of MT− vs. MTx and in MT+ vs. MTx. We expected that the two opposite MTs produced distinctive molecules, including at least an MT+ specific pheromone and an MT− specific pheromone. 

**Figure 4:**
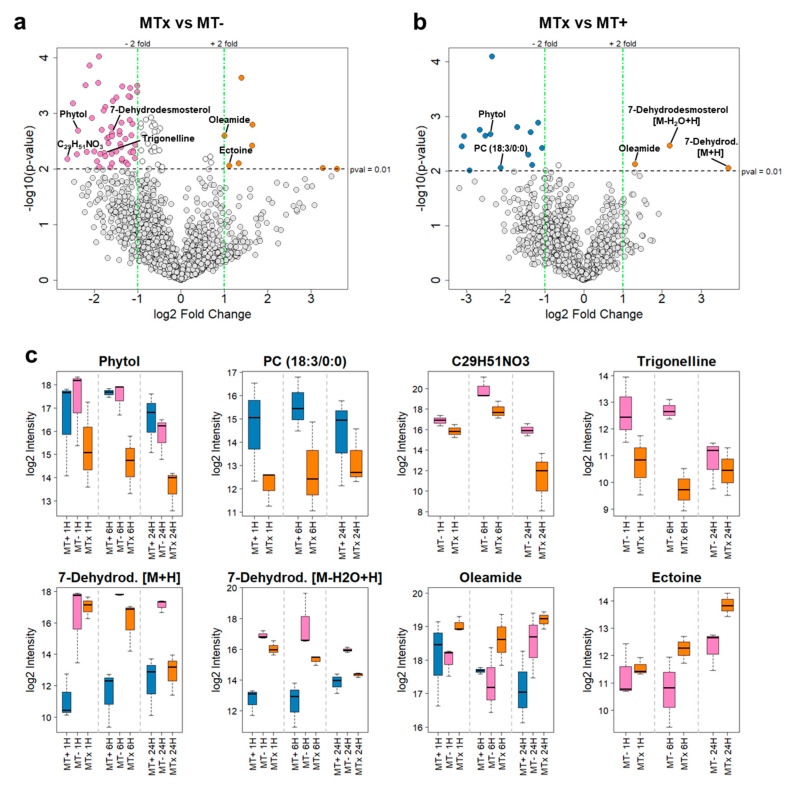
Statistical analysis of metabolite accumulation in different sample types using DCM as extraction solvent. (**a**) Volcano plot of the differences in metabolite accumulation between MT− and MTx for the three time points. Data statistical significance (*p*-value in a log_10_ scale) is plotted versus fold change in a log_2_ scale. The horizontal dashed line shows a *p*-value of 0.01, and the two vertical dashed lines separate features having a fold change of 2. Dots on the left and right of the dashed vertical lines and above the horizontal line represent highly abundant features in MT− (pink; fc < −2) and in MTx (orange; fc > 2), respectively. Grey dots represent features that are statistically insignificant (*p*-value > 0.01), and/or have an insignificant fc. (**b**) Volcano plot of the differences in metabolites accumulation between MT+ and MTx for the three time points. Features more abundant in MT+ are depicted in blue and features more abundant in MTx are depicted in orange. (**c**) Box plots of log_2_ scaled intensities of annotated/identified metabolites at 1 h, 6 h and 24 h with high fold change values between MT− vs. MTx and MT+ vs. MTx with *p*-value < 0.01. (For 7-Dehydrod. [M + H], differences between MT− vs. MTx are not statistically significant). 7-Dehydrod. = 7-Dehydrodesmosterol.

The alterations in metabolite abundances between MTx vs. MT− and between MTx vs. MT+ were visualized with volcano plots (Figure 4a,b), that depict statistical significance (*p*-value in a log_10_ scale) versus the fold change (fc) in a log_2_ scale. This evaluation revealed significant variations in 63 features for MT− vs. MTx and in 17 features for MT+ vs. MTx. 55 of these features had higher abundance in MT− compared to MTx, while 14 were found to be more abundant in MT+ compared to MTx.

Dysregulated features were tentatively identified wherever possible based on MS/MS analysis and spectral databases matching (Figure 4c and Table 1). However, the chemical complexity or the low intensity of the majority of these features, leading to absence of MS/MS spectra, did not allow a complete annotation. Notably, the levels of phytol were found to be at least 4 times lower in MTx with respect to the other two MT groups at every harvesting time point. A lyso-PC(18:3/0:0) showed a higher intensity in MT+ vs. MTx at every time point, whereas this feature was found to be non-significant in the comparison between MT− vs. MTx (*p* value higher than 0.01).

A feature with an exact neutral mass of 461.3869 Da and a putative sum formula of C_29_H_51_NO_3_ was found to be upregulated in MT− vs. MTx, however its chemical structure could not be assigned. It resulted to be the one with the highest fold change (log_2_fc = −2.61) in the MT− vs. MTx analysis. A similar behavior was observed for trigonelline (log_2_fc = −1.77).

Oleamide and ectoine were, among the identified compounds, those with higher accumulation in MTx at every time point. Ectoine showed a steadily increase in intensity over time.

Finally, two pseudomolecular ions of *m/z* = 383.3309 Da (for [M + H]^+^) and *m/z* = 365.3204 Da (for [M − H_2_O + H]^+^) and identical retention times raised our interest, since one exhibited the highest (approximately 13-fold) upregulation in the comparison between MT+ vs. MTx. Moreover, these features resulted as the highest dysregulated ones in the comparison between the two MT of opposite sex (MT+ vs. MT−, Figure S3), since the levels of the [M + H]^+^ and of the [M − H_2_O + H]^+^ pseudomolecular ions were 45- and 14-fold higher in the MT− compared to the MT+ (log_2_fc [M + H]^+^ = 5.50 and log_2_fc [M − H_2_O + H]^+^ = 3.79), respectively.

Both features had similar MS/MS spectra that were part of the steroids subnetwork in the MN. Their intensity increased over time in the MT+ population, while it decreased over time in the MT− and MTx populations. The molecular formula of the neutral molecule was determined as C_27_H_42_O. The compound was tentatively identified as 7-dehydrodesmosterol, based on the MN structural similarities that placed the aforementioned ions in the steroids subfamily, followed by a fragmentation pattern analysis and a comparative metabolomics approach, where MS/MS spectra were matched with specific sterol standard molecules having the same molecular formula (Figure S4).

## 3. Discussion

In this study we investigated the chemical diversity of two *P. multistriata* strains using a LC–MS untargeted metabolomics approach. Moreover, we explored changes in the metabolic profile of cells during the process of sexual reproduction over the first 24 h, identifying a small subset of well-studied compounds commonly found in animals or plants, in addition, expectedly, to a larger fraction of poorly annotated features.

An effective extraction method for the HPLC-MS/MS screening was assessed by the sequential use of three solvent systems (DCM, DCM:MeOH and MeOH) of increasing polarity, which enabled us to capture solvent-specific metabolites. The majority of these was extracted with the first solvent used, DCM; this result may not only be due to a higher efficiency of DCM as an extraction solvent, but it also reflects the sequential order of extractions. A sequential order was chosen to maximize the number of extracted metabolites from a limited amount of biomass, which was too small for investigating three parallel extractions or alternative orders of sequential extractions. Metabolites extracted with the other two solvents used subsequently were lower in number, but they allowed to expand the repertoire of molecules obtained in the study. Whereas all three solvent systems were able to detect a common core metabolome, i.e. ca. 12% of nodes (Figure 3b), two thirds of the nodes (approximately 67%) were extracted by only one solvent. These metabolites may have been completely missed in the present screening if any of these solvent systems would have been omitted. The study, thus, demonstrates the improvement in detectable molecular diversity that can be achieved by sequential extractions with different solvents.

A full understanding of the mechanisms at play is hampered by the limited annotation of features to structurally defined metabolites. While 2408 high quality features were obtained in the overall set of experiments (some of them redundant, as they stem from the same metabolite), merely 70 metabolites could be safely identified. The lack of annotation reflects the probably most severe technical shortcoming in today’s untargeted metabolomics studies [23,24,25]. This issue is exacerbated in rarely studied organisms like diatoms, because comprehensive databases, reference datasets and reference standards, as established for human metabolomics, are missing.

To alleviate this, the use of additional separation techniques like gas chromatography (GC) or hydrophilic interaction chromatography (HILIC), and the use of negative ionization are suited to capture additional parts of the metabolome, which were not detected in the present Reversed-Phase (RP) LC/MS approach. For example, the analytical method used in this study is not well-suited for the detection of lipids such as triglycerides and of highly polar sugars.

GC-MS data are expected to complement information particularly on primary metabolism, as the mass spectra can be annotated well with the help of highly curated databases.

In the present study, MN proved particularly valuable to classify non-identified features, based on their structural similarities to known metabolites. Thus, for poorly studied organisms that may express rare or even unknown metabolites, a comparison of MS/MS fragmentation similarity is a viable option to capture the number of different molecular families, and to assign unknowns at least to a chemical class.

Most of the metabolomics studies in diatoms are focused on the changes associated to specific abiotic parameters, such as nutrient availability (nitrogen limitation, iron starvation) [26,27,28] and salinity variation [29]. These studies reveal that diatoms can efficiently and rapidly alter their metabolic pathways and abundances to adapt to the changing environment. In the last decade, only few studies were published on metabolic profiles of diatoms testing their global response to biotic factors; in particular investigated species included *Cocconeis scutellum* [30], *Skeletonema marinoi* [15,31], *Seminavis robusta* [9], *Chaetoceros calcitrans* [32] and *Chaetoceros tenuissimus* [33]. However, we are still very far from having a comprehensive picture of these organisms and of the metabolic hierarchies supporting the function of individual species. Moreover, despite the interest of the scientific community in *Pseudo-nitzschia*, this is the first broad metabolomics analysis of a species belonging to this important toxin-producing genus, to the best of our knowledge.

The data generated in *P. multistriata* broaden our knowledge of the chemical diversity in this group of microalgae. Our analysis allowed to identify 70 known metabolites, including fatty acids, steroids, eicosanoids, amides, lysophosphatidylcholines and lipophosphoglycans, metabolites from Tyr and Ala metabolic pathways and, expectedly, the toxin domoic acid. Moreover, 46 additional features were assigned to chemical classes based on their MS/MS similarities with known compounds; such features, not represented in our libraries of compounds, could not be assigned by conventional database searches. The rest of the features were neither dereplicated by computational methods, nor attributed to a specific chemical class based on their MS/MS patterns. Collectively these findings indicate that *P. multistriata* produces unusual metabolites that are not represented in reference databases, some of which might even belong to novel chemical scaffolds.

The process of sexual reproduction has been extensively explored in this species in terms of timing, morphological, physiological and gene expression changes associated to the different phases. The MT− in our study was the one exhibiting the highest number of specific nodes. This matches previous observations at the transcriptome level: the MT− was the one exhibiting the highest number of differentially expressed genes compared to MT+ [20] (Annunziata et al., in preparation).

A very striking phenomenon occurring in *P. multistriata* is the prolonged growth arrest of the cells after strains of opposite MTs have been mixed, lasting up to five days after the start of sexual reproduction [34] and evident already at six hours [20]. Growth arrest occurs in a condition of nutrient repletion and affects all cells in the cross, not only those which will effectively turn into gametes. This arrest is accompanied by very prominent gene expression changes, as revealed by RNA-seq studies of cells exposed to the medium of the opposite MT [20] or mixed in a cross (Annunziata et al., in preparation), compared to vegetatively growing cultures. 

Gene expression data point to a general shut down of the main metabolic processes, including a downregulation of photosynthesis, the downregulation of nutrient transport and of lipid biosynthesis.

In this context, it is interesting to note that one of the most downregulated metabolites in the cross sample is phytol, a constituent of chlorophyll and often a product of its degradation, possibly reflecting a reduction of the photosynthetic apparatus in the arrested cells.

Some of the changes observed in the metabolite content could be indeed linked to the growth arrest rather than to the specific processes related to sexual reproduction, such as migration of opposite MTs towards each other, to gametangia pairing or to meiosis and gamete appearance. To tell apart the different contributions, a comparative experiment where cell growth is arrested with other methods (e.g. by the addition of inhibitors of cells division) should be designed.

The levels of oleamide increase in the cross compared to the two MTs in monoculture. Oleamide is a fatty acid amide derived from oleic acid, produced in animals, plants as well as in microalgae, that interacts with a variety of membrane receptors, such as GABA(A) and 5-HT(7), and has cannabinoid-like actions [35]. Interestingly, oleamide has been shown to be effective in reducing biofouling: oleamide treated surfaces become slippery and the biofoulers, including benthic diatoms, attach weakly [36]. *P. multistriata* is a pennate species and despite being planktonic, it has retained the ability to slide on surfaces due to the presence of a raphe, a slit in the siliceous frustule through which mucus is secreted to promote movement. *P. multistriata* cells become immediately more motile when placed in a cross, possibly because each cells starts actively looking for a partner cell to pair with [37]. Increased oleamide secretion could promote cell motility or could be related to the process of gametangia detaching from the frustules when gametes are produced.

Another metabolite which changed specifically in the cross compared to the MT− monoculture is ectoine. This is a known bacterial compound required for osmoregulation, and since the cultures used in this study were not made axenic, it is possible that its detection is due to the presence of one or more ectoine producing marine bacteria, deriving specifically from the MT− culture. Its steady increase over time could reflect a higher bacterial growth in the cross, in which diatom cells, as just mentioned, are arrested and less metabolically active, or a stimulation of ectoine synthesis due to the mixing with the MT+ culture and its associated bacterial population. Interestingly, a recent study provides evidence of ectoine synthesis in axenic microalgal cultures and reports the presence of putative ectoine biosynthetic genes in diatoms [38]. Therefore, an alternative hypothesis is that *Pseudo*-*nitzschia* cells do synthetize ectoine. In the *P. multistriata* genome, a TBLASTN search with the protein sequence for the *EctA*, *EctB,* and *EctC* biosynthetic genes from *Halorhodospira halochloris* retrieved entries for the first two genes only, however a reciprocal BLASTP against the *Halorhodospira halochloris* protein database did not confirm homology (data not shown), suggesting that the identification of the putative ectoine biosynthetic genes in diatoms requires further investigations.

Trigonelline, an alkaloid found in many plant species, and a metabolite with a putative sum formula of C_29_H_51_NO_3_ are downregulated in the cross compared to MT− (Figure 4c and Table 1), while these features are not statistically significant (*p* > 0.01) in the comparison MT+ vs. MTx. Similarly, a lyso-PC with a remaining 18:3 chain is downregulated in the cross compared to MT+, while not being significantly affected in MT− vs. MTx.

These changes could be due to a real reduction of the synthesis of these compounds when cells engage in sexual reproduction, or they could simply be the effect of the dilution of the producing culture with the non-producing culture of opposite MT. At least for the putative C_29_H_51_NO_3_, the downregulation in comparison with MTx seems to increase over time, making the hypothesis of a dilution effect less likely. With only two strains investigated here, it is impossible to tell whether a compound found in only one of the two MTs reflects MT specificity, or natural strain variability. Testing for the presence and regulation of these compounds in other independent MT+ and MT− strains will clarify whether trigonelline, C_29_H_51_NO_3_ and PC (18:3/0:0) are truly specific to the MT. If, by enlarging the number of strains analyzed, they are found in both MTs, a cross could be set up to verify whether their downregulation still occurs.

The concentration of 7-dehydrodesmosterol in the cross is increased compared to the MT+ and decreased compared to the MT−. Moreover, 7-dehydrodesmosterol is an intermediate that leads to the production of cholesterol. Sterols containing 27, 28, and 29 carbon atoms are commonly found in diatoms, their biosynthetic pathways appear to contain features of both plant and fungal pathways [39]. It is currently challenging to link changes in this metabolite with specific biosynthetic genes in *P. multistriata* and the significance of this finding remains to be understood.

In this first exploration of *P. multistriata* cells chemical composition, we focused on changes occurring in the endometabolome. A complementary study will foresee the analysis of compounds secreted by the cells (i.e. the exometabolome) in a similar setup. Such an effort should yield information of *P. multistriata* sex pheromones. Given the scarce information for diatoms and the natural high variability of pheromones, a prediction of the chemical nature of the *P. multistriata* pheromones is very difficult, and the molecules involved could be of different type. Gene expression analyses have identified a set of genes which, in one MT, respond to the addition of the medium conditioned by the other MT [40]. The activation of these genes, detected by qPCR, could be used as a readout in a bioassay-guided fractionation of the medium, to pursue identification of the pheromones in the future.

## 4. Materials and Methods

### 4.1. Strains and Cultivation Procedure

Strains used for this experiment were LV168 (MT+) and LV123 (MT−) [22], both with an average cell length of about 30 μm. Artificial sea water was used as medium. The experiment was set up in a culture chamber at 18 ± 2 °C with a photoperiod of 12:12 h light:dark and an irradiance of 130 μmol photon m−2 s−1. 14 liters culture for each MT was set up starting from a cell concentration of 10,000 cells/mL. When cell density reached a concentration between 140,000 and 160,000 cells/mL, 500 mL of the two cultures were mixed in a glass flask (cross), while 1 L of each monoculture was moved in a separate glass flask to serve as control. Triplicates were prepared for each sample at each time point. Samples were collected at 1 h, when cells of the two MTs have detected the presence of the opposite MT and are reacting to the presence of primary pheromones, at 6 h, when cells of opposite MTs are searching for a partner and start the mating process, and 24 h, when gametes have appeared. At each sampling time, 1 l of each culture (MT+, MT−, and Cross), in triplicate, were gently filtrated using Millipore^®^ Stericup™ (Darmstadt, Germany) filters with 0.22 µm pores, cells were recovered from the filter in a small volume and were then centrifuged at 3220 g, for 20 min, at 18 °C. The pellets were frozen with liquid nitrogen and stored at −80 °C, and were then lyophilized to obtain dry biomass.

The experimental set up described above and used in the present study is schematized in Figure 1a. The growth of MT+, MT−, and Cross was followed with separate controls not only for the 24 h of the experiment, but also for the following days to make sure that the whole process of sexual reproduction (which lasts a few days and ends with appearance of a new generation of F1 cells) was completed successfully. In the control plate, inspecting the plate daily for 5 days, we could see the expected behavior of the cross (almost no growth, appearance of sexual stages) and of the parental cells (increase in the cell density).

### 4.2. Extraction of Endo-Metabolites

Freeze-dried biomass was first disrupted using a mortar, and then 1.5 mL of dichloromethane (DCM) was added to the pellets in a 2-mL Eppendorf tube. The mixture was homogenized by shaking, and complete disruption of cell membranes was achieved via sonication (Sonorex Digiplus, BANDELIN electronic, Berlin, Germany) in an ice-cold water bath for 10 min.

Subsequently, the remaining pellets were sequentially extracted with 1:1 dichloromethane:methanol (DCM:MeOH), followed by methanol (MeOH), using the same procedure as described above.

Cell debris was removed from all extracts by centrifugation at 4 °C and 11,000× *g* for 12 min, and the supernatants containing endo-metabolites (i.e., metabolites extracted from cell pellets) were collected and transferred by pipetting to clean, pre-weighted Eppendorf tubes. Supernatants were dried in a centrifugal evaporator (Refrigerated CentriVap Concentrator equipped with −50 °C CentriVap Cold Trap, Labconco, Kansas City, MO, USA) at 20 °C and full vacuum until complete dryness; dried extracts were weighted. 

Samples were reconstituted in acetonitrile (for DCM extractions) or in 1:1 acetonitrile:water (for DCM:MeOH and MeOH extractions) at a concentration of 10 mg/mL. Reconstitution solvents contained 0.8 mg/Lof naproxen (Sigma-Aldrich, Taufkirchen, Germany), which was used as internal standard to monitor chromatographic behavior and normalize the measurements. Naproxen is not expected to be produced by the investigated organism.

80 µL of the reconstituted samples were transferred to HPLC vials equipped with glass inlets for LC-MS analysis. Equal volumes of each of the samples extracted with the same solvent system were combined to yield a pooled sample, which was used for further analysis.

All solvents used were Baker Analyzed™ Ultra LC-MS grade (Fisher Scientific, Schwerte, Germany).

### 4.3. UPLC-ESI-QToF-MS/MS Untargeted Metabolomic Profiling

Reconstituted extracts were analyzed by ultra-high-performance liquid chromatography–tandem mass spectrometry (UPLC-ESI-QToF-MS/MS). An untargeted metabolomics approach was used for an unbiased analysis of the endo-metabolomes (Figure 1b). Five µL of each sample were subjected to chromatographic separation using a UltiMate 3000 RS (Thermo Scientific Dionex, Waltham, MA, USA) UPLC system, equipped with a Kinetex C18 reversed phase column (1.7 µm, 150 × 2.1 mm from Phenomenex, Aschaffenburg, Germany) kept at 40 °C, with a flow rate of 300 µL/min. A gradient elution with water (+0.1% *v/v* formic acid) as eluent A and acetonitrile (+0.1% *v/v* formic acid) as eluent B, was run as follows: 1% B for 0 to 2 min, linear gradient from 1% B to 100% B from 2 to 20 min, hold 100% B until 25 min and linear gradient from 100% B to 1% B from 25 to 30 min.

Column eluates were analyzed in positive electrospray ionization mode on a Bruker maXis HD QToF mass spectrometer equipped with an Apollo II electrospray source (Bruker, Bremen, Germany). Raw data were acquired in full scan mode (50–1500 Da) in a data dependent MS/MS mode, performing collision-induced fragmentation of the five most abundant ions in each MS scan and using of Bruker’s “smart exclusion” functionality to minimize multiple fragmentation of the same ion. The collision energy was ramped from 80% to 200% of the default auto-MS/MS collision energy in order to get more information rich spectra.

The samples preparation and chromatographic separation carried out and described above are not optimal for the detection of lipids, which, containing polar heads and nonpolar fatty acyl chains, have distinct physicochemical properties and thus require specific methods of analysis; however, the present method was put in place to the aim of detecting the largest number of compounds possible.

### 4.4. Data Processing and Metabolomics Analysis

#### 4.4.1. Data Preprocessing

Raw LC-MS/MS data were lock mass calibrated and converted to mzXML files using Bruker DataAnalysis (version 4.2, Bruker, Bremen, Germany) and Bruker Compass Xport (version 3.0.13, Bruker, Bremen, Germany) softwares.

Detection of chromatographic peaks, retention time correction, normalization on the internal standard and filtering of detected features (retention time – *m/z* pairs) was carried out using R (version 3.6.1, R Foundation for Statistical Computing, Vienna, Austria) in the RStudio environment [41,42] (version 1.2.1578, PBC, Boston, USA), mainly with the package xcms [43]. This resulted in a processed feature table (available in the Supplementary Materials, file “Processed feature table”), which was used for statistical analysis, as described in the following paragraph.

#### 4.4.2. Statistical Analysis

Principal component analysis (PCA) was performed on the global dataset in order to provide an unbiased overview of individual samples and to reveal patterns in the data and relationships between groups.

To highlight alterations in metabolite accumulation driven by the different sample types, univariate analysis was performed (volcano plots). Data statistical significance (*p*-value in a log_10_ scale) was plotted in function of the fold change (fc) in a log_2_ scale, between MT− vs. MTx and between MT+ vs. MTx. The intensity of identified metabolites with significant variations was visualized with box plots. The R packages metabolomics [44], muma [45], RColorBrewer [46], ggplot2 [47], dplyr [48], ropls [49], and venneuler [50] were used for statistical analysis.

#### 4.4.3. Metabolite Annotation/Identification 

Features were searched first against our in-house library, built with analytical standards from the MSMLS—Mass Spectrometry Metabolite Library of Standards (IROA Technologies, Bolton, MA, USA) as well as a number of individually bought compounds from Sigma-Aldrich (Taufkirchen, Germany). Identification was confirmed by matching the precursor mass, retention time and MS/MS spectrum values to the available standards.

When standards were not available in the in-house library, known metabolites annotation was carried out by searching their mass values and comparing their MS/MS spectra with online spectral databases, such as METLIN (https://metlin.scripps.edu), the human metabolome database (http://www.hmdb.ca) and GNPS (https://gnps.ucsd.edu/), for putative metabolite identification.

Moreover, SIRIUS4, a software framework for de-novo identification of metabolites, was used to confirm the attribution of some interesting metabolites; this tool analyzes isotope patterns in fragments for the deduction of molecular formula and identification of the compound [51,52,53].

#### 4.4.4. Molecular Networking

The mzXML files were uploaded to the Global Natural Products Social Molecular Networking (GNPS, [54] http://gnps.ucsd.edu) online tool, and a molecular network (MN) was generated through the classical online workflow available from GNPS website.

Data were filtered by removing all MS/MS fragment ions within +/− 17 Da of the precursor *m/z*. The precursor ion mass tolerance was set to 0.005 Da and a MS/MS fragment ion tolerance of 0.02 Da. A network was then created where edges were filtered to have a cosine score above 0.7 and more than 6 matched peaks. Edges between two nodes were kept in the network only if each of the nodes appeared in each other’s respective top 10 most similar nodes.

Finally, the maximum size of a molecular family was set to 100, and the lowest scoring edges were removed from molecular families until the molecular family size was below this threshold. The spectra in the network were then searched against GNPS’ spectral libraries. The library spectra were filtered in the same manner as the input data. All matches kept between network spectra and library spectra were required to have a score above 0.7 and at least 6 matched peaks.

The analysis of the obtained MN was conducted through Cytoscape software [55] (version 3.7.1, San Diego, USA).

The features identified by GNPS where matched against the processed feature table by exact mass and retention time; the GNPS annotation was maintained when the two features (from GNPS and from the processed feature table) were matching with an m/z tolerance of ±8 ppm and a retention time constrain of ±25 s.

### 4.5. Data Accessibility

Raw measurements files are available on MetaboLights. Study Identifier: MTBLS1714.

Molecular networking data are available from GNPS website (https://gnps.ucsd.edu/ProteoSAFe/status.jsp?task=b7638ba3d90b422d97614972ae6116a9).

Computational code written for this study is available upon request.

## 5. Conclusions

This exploratory metabolomics study allowed us to obtain a snapshot of the chemical diversity detectable in the diatom *Pseudo-nitzschia multistriata*, a member of a species-rich genus of global importance, well known because of its ability to form toxic algal blooms. The comparison of three different conditions at different time points revealed the static and the dynamic parts of metabolism occurring during sexual reproduction. While linking metabolite signals (‘features’) to chemical structures and to their biosynthetic genes is still a challenge in these relatively poorly explored systems, our finding of a reduction in phytol in the cross matches the observation of a general downregulation of photosynthesis during sexual reproduction (Annunziata et al., in preparation). Other regulated metabolites offer starting points to better understand the mechanisms controlling one of the most delicate phases of the diatom life cycle. While this is the first broad metabolomics analysis in *Pseudo-nitzschia*, further studies with complementary analytical techniques and other experimental setups (e.g. directed to exo-metabolites or volatiles) are required for a more comprehensive grasp of metabolism in diatoms.

## Figures and Tables

**Table 1:** Annotated/identified metabolites, extracted with DCM, which were found to have a significant fold change (i.e., *p* < 0.01 and −1 < log_2_ fc > 1) when comparing MTx vs. MT+ and MTx vs. MT−. The log_2_ fc values of the most dysregulated features are printed in bold.

Metabolite	*m/z* [Da]	r.t. med [min]	log_2_ fc MTx vs. MT+	log_2_ fc MTx vs. MT−
Phytol	319.2974	22	−2.39	−2.37
PC (18:3/0:0)	518.3244	16	−2.13	Not significant (*p* > 0.01)
C_29_H_51_NO_3_	462.3944	15	Not significant (*p* > 0.01)	**−2.61**
Trigonelline	138.0551	1	Not significant (*p* > 0.01)	−1.77
7-Dehydrodesmosterol [M + H]	383.3309	21	**3.70**	Not significant (*p* > 0.01)
7-Dehydrodesmosterol [M − H_2_O + H]	365.3204	21	2.20	−1.59
Oleamide	282.2794	19	1.31	1.00
Ectoine	143.0817	1	Not significant (*p* > 0.01)	1.12

## Supplementary Materials

The following are available online at https://www.mdpi.com/1660-3397/18/6/313/s1. Figure S1: Box plots of log_2_ scaled intensities of domoic acid in the different samples (MT+ blue, MT− pink, MTx orange) at 1h, 6h, and 24h, from MeOH extraction. Figure S2: Average intensities of features (MS1 level). Left panel: Plot of intensity vs. harvesting time. Right panel: Average intensities of features belonging to a molecular weight category. Features from all three solvent extractions are considered. Mating types are MT+ (male, in blue), MT− (female, in pink), and MTx (cross, in orange). Figure S3: Volcano plot of the differences in metabolites accumulation between MT- and MT+. Data statistical significance (*p*-value in a log_10_ scale) is plotted versus fold change in a log_2_ scale. The horizontal dashed line shows a *p*-value of 0.01, and the two vertical dashed lines separate features having a fold change of 2. Dots on the left and right of the dashed vertical lines and above the horizontal line represent high abundant features in MT- (pink; fc > 2) and in MT+ (blue; fc < −2). Grey dots represent features that are statistically insignificant (*p*-value > 0.01), and/or have an insignificant fc. 7-Dehydrod. = 7-Dehydrodesmosterol; these are the only annotated features among those statistically significant. Figure S4: MS/MS spectra of 7-Dehydrodesmosterol in a sample (top and middle) and in the standard (bottom). Figure S5: High resolution version of the MN in Figure 3a. Table S1: Identification (i.e., *m/z* and r.t. match, in bold) and annotation (i.e., *m/z* and MS/MS spectral match) of known metabolites, based on in-house libraries, online databases and MS/MS fragmentation similarities. Table S2: Indication of the chemical class of features based on similarity of the fragmentation pattern. Processed feature table: Full list of 2408 high-quality features obtained for the whole experiment.
