# Supplementary material for: A Metabolomics Exploration of the Sexual Phase in the Marine Diatom Pseudo-nitzschia multistriata
Source: Mar Drugs. 2020 Jun 14;18(6):313. doi: 10.3390/md18060313 (PMC7345340; doi:10.3390/md18060313)
Supplement: Supplementary file 1 [file marinedrugs-18-00313-s001.zip › marinedrugs-831481-supplementary.docx]

Supporting Information

A metabolomics exploration of the sexual phase in the marine diatom Pseudo-nitzschia multistriata

Federica Fiorini, Camilla Borgonuovo, Maria Immacolata Ferrante and Mark Brönstrup

Table S1. Identification (i.e. m/z and r.t. match, in bold) and annotation (i.e. m/z and MS/MS spectral match) of known metabolites, based on in-house libraries, online databases and MS/MS fragmentation similarities.

| **m/z**  **[Da]** | **r.t. med [min]** | **Annotation** |
| --- | --- | --- |
| 104.1072 | 1 | **Choline** |
| 116.0708 | 1 | **Proline** |
| 118.0864 | 1 | **Betaine** |
| 120.0809 | 3 | **Phenylethanolamine** |
| 130.0865 | 1 | **L-Pipecolic acid** |
| 132.1021 | 2 | Isoleucine |
| 136.0759 | 2 | 2-Phenylacetamide |
| 137.046 | 3 | **Hypoxanthine** |
| 138.0551 | 1 | **Trigonelline** |
| 143.0817 | 1 | Ectoine |
| 146.1178 | 1 | **Acetylcholine** |
| 148.0607 | 1 | **L-Glutamic acid** |
| 150.0585 | 1 | **L-Methionine** |
| 162.1127 | 1 | **L-Carnitine** |
| 165.0549 | 2 | p-hydroxyphenyllactic acid |
| 166.0865 | 3 | **L-phenylalanine** |
| 182.0815 | 2 | **Tyrosine** |
| 188.0709 | 6 | N-Methyl-L-tryptophan |
| 195.0879 | 7 | **Caffeine** (std.) |
| 197.1175 | 9 | Loliolide |
| 203.0529 | 1 | Glucose |
| 204.1233 | 1 | Acetyl-DL-carnitine |
| 205.0975 | 6 | L-Tryptophan |
| 215.0165 | 1 | Isocitric acid |
| 220.1123 | 16 | N-Phenyl-2-naphthylamine |
| 223.0968 | 13 | Diethyl phthalate (contaminant) |
| 229.2164 | 18 | Isomyristic acid |
| 235.1656 | 1 | **Betaine** |
| 237.2215 | 18 | Palmitelaidic acid |
| 255.2321 | 18 | Palmitoleic Acid |
| 256.2637 | 19 | Palmitamide |
| 257.2478 | 19 | **Hexadecanoic acid** |
| 258.1104 | 1 | **sn-Glycero-3-phosphocholine** |
| 260.1859 | 8 | Hexanoyl-L-carnitine |
| 265.2527 | 19 | 9-Octadecenamide, (Z)- |
| 269.0884 | 3 | **Inosine** |
| 273.2578 | 19 | (-)-Ent-copalol |
| 277.2165 | 17 | 13-HoTrE |
| 279.2321 | 18 | 9(10)-EpOME |
| 282.2794 | 19 | Oleamide |
| 283.2634 | 17 | 3-Hydroxyoctadecanoic Acid |
| **m/z**  **[Da]** | **r.t. med [min]** | **Annotation** |
| 285.2216 | 18 | Abieta-8(14),9(11),12-triene-7,18-diol |
| 285.2791 | 21 | Stearic acid |
| 297.279 | 19 | Phytomonic acid |
| 301.2165 | 15 | 17(18)-EpETE |
| 303.2321 | 18 | 20-HETE |
| 305.2478 | 18 | 15-OxoEDE |
| 307.0838 | 1 | **Glutathione oxidized** |
| 307.2634 | 19 | 15-HEDE |
| 311.2584 | 16 | Monopalmitolein (9c) |
| 312.1445 | 7 | Domoic acid |
| 318.3006 | 13 | Phytosphingosine |
| 319.2974 | 21 | **Phytol** |
| 329.243 | 21 | **Docosahexaenoic acid** |
| 338.3421 | 22 | 13-Docosenamide, (Z)- |
| 348.0707 | 1 | **Adenosine 5'-monophosphate** |
| 353.269 | 17 | Monolinolenin (9c,12c,15c) |
| 365.1058 | 1 | **Palatinose** |
| 365.3204 | 21 | **7-Dehydrodesmosterol** |
| 367.3362 | 23 | Zymosterol |
| 369.1253 | 17 | Tricresylphosphate (contaminant) |
| 371.1016 | 19 | Cyclopentasiloxane, decamethyl- (contaminant) |
| 383.3309 | 21 | **7-Dehydrodesmosterol** |
| 387.1806 | 13 | 1-O-Sinapoyl-beta-D-glucose |
| 397.3293 | 20 | **Vitamin D2** |
| 399.3257 | 19 | (3β)-3,26-Dihydroxycholest-5-en-7-one |
| 413.2666 | 21 | **Dioctyl Phthalate** (contaminant) |
| 415.2118 | 14 | Niranthin |
| 425.215 | 17 | TyrSerArg |
| 446.186 | 12 | **Glipizide** (std.) |
| 476.2774 | 15 | LPE(18:3) |
| 490.2931 | 15 | LPC(14:0) |
| 492.3087 | 16 | LPC(16:1) |
| 494.3244 | 18 | LPC(16:0) |
| 507.272 | 15 | LPG(18:3) |
| 518.3244 | 16 | LPC(18:3) |
| 526.293 | 16 | LPE(22:6) |
| 581.3989 | 19 | Fucoxanthin |
| 659.4304 | 19 | Fucoxanthin |
| 804.5464 | 21 | **Dioctyl phthalate** (contaminant) |

Table S2. Indication of the chemical class of features based on similarity of the fragmentation pattern.

| **m/z**  **[Da]** | **r.t. med [min]** | **MN chemical classification** |
| --- | --- | --- |
| 137.0435 | 1 | Trigonelline subfamily |
| 137.0599 | 10 | Trigonelline subfamily |
| 181.0263 | 2 | Tyr and Ala metab. Subfamily |
| 183.0848 | 2 | Tyr and Ala metab. Subfamily |
| 231.1746 | 13 | Fatty acids and amides |
| 233.1902 | 14 | Fatty acids and amides |
| 235.206 | 17 | Fatty acids and amides |
| 249.1852 | 16 | Fatty acids and amides |
| 251.2008 | 17 | Fatty acids and amides |
| 267.1229 | 14 | 1-O-Sinapoyl-beta-D-glucose subfamily |
| 275.2008 | 17 | Eicosanoids |
| 283.1179 | 12 | 1-O-Sinapoyl-beta-D-glucose subfamily |
| 285.2791 | 21 | Saturated fatty acids |
| 301.214 | 18 | Eicosanoids |
| 307.1907 | 11 | Fucoxanthin subfamily |
| 313.2352 | 16 | Fatty acids and amides |
| 315.2896 | 19 | Fatty acids and amides |
| 323.222 | 13 | Fatty acids and amides |
| 329.269 | 15 | Fatty acids and amides |
| 369.1699 | 13 | 1-O-Sinapoyl-beta-D-glucose subfamily |
| 375.2509 | 17 | TyrSerArg subfamily |
| 399.2533 | 15 | Fucoxanthin subfamily |
| 403.1756 | 13 | 1-O-Sinapoyl-beta-D-glucose subfamily |
| **m/z**  **[Da]** | **r.t. med [min]** | **MN chemical classification** |
| 404.2071 | 13 | 1-O-Sinapoyl-beta-D-glucose subfamily |
| 404.3164 | 13 | 1-O-Sinapoyl-beta-D-glucose subfamily |
| 415.1755 | 14 | 1-O-Sinapoyl-beta-D-glucose subfamily |
| 485.2749 | 13 | Fatty acids and amides |
| 489.2274 | 13 | 1-O-Sinapoyl-beta-D-glucose subfamily |
| 489.34 | 16 | LysoPCs |
| 492.3087 | 16 | LysoPCs |
| 494.3244 | 17 | LysoPCs |
| 505.2222 | 13 | 1-O-Sinapoyl-beta-D-glucose subfamily |
| 515.3192 | 17 | LysoPCs |
| 518.3244 | 17 | LysoPCs |
| 520.3399 | 18 | LysoPCs |
| 529.2679 | 14 | Fatty acids and amides |
| 549.2366 | 13 | Fatty acids and amides |
| 550.314 | 13 | LysoPCs |
| 551.2522 | 13 | Fatty acids and amides |
| 557.4565 | 18 | Fatty acids and amides |
| 568.3398 | 17 | LysoPCs |
| 605.4564 | 18 | Eicosanoids |
| 641.42 | 20 | Fucoxanthin subfamily |
| 643.2924 | 19 | Fucoxanthin subfamily |
| 659.287 | 20 | Fucoxanthin subfamily |
| 771.5018 | 22 | LysoPCs |


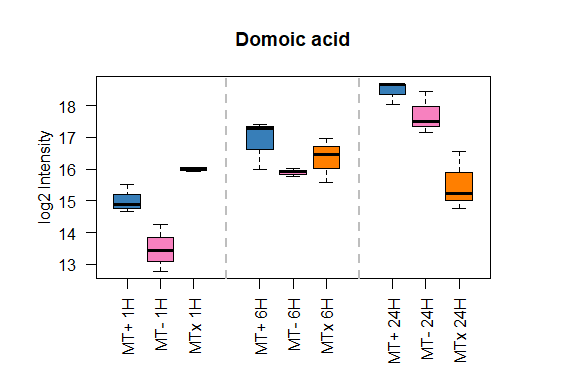


**Figure S1**. Box plots of log2 scaled intensities of domoic acid in the different samples (MT+ blue, MT- pink, MTx orange) at 1h, 6h and 24h, from MeOH extraction.


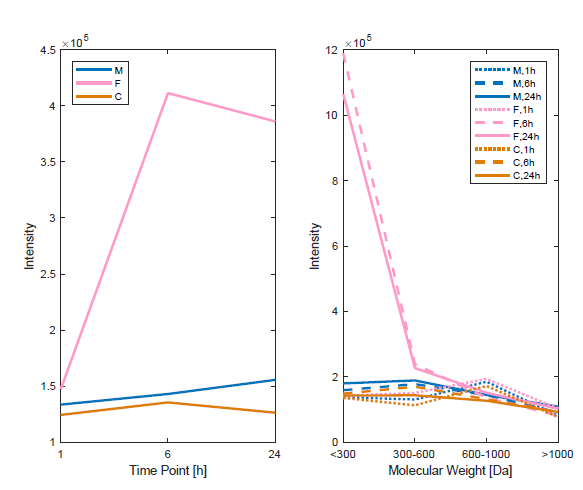


**Figure S2.** Average intensities of features (MS1 level). Left panel: Plot of intensity vs. harvesting time. Right panel: Average intensities of features belonging to a molecular weight category. Features from all three solvent extractions are considered. Mating types are MT+ (male, in blue), MT- (female, in pink) and MTx (cross, in orange).


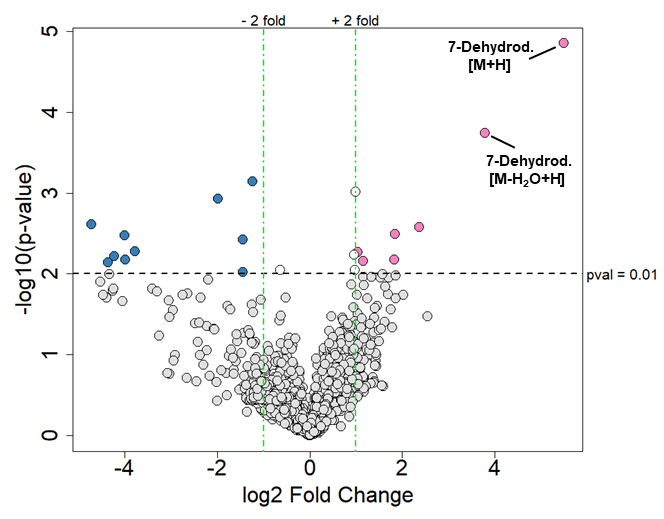


**Figure S3.** Volcano plot of the differences in metabolites accumulation between MT- and MT+. Data statistical significance (p-value in a log10 scale) is plotted versus fold change in a log2 scale. The horizontal dashed line shows a p-value of 0.01, and the two vertical dashed lines separate features having a fold change of 2. Dots on the left and right of the dashed vertical lines and above the horizontal line represent high abundant features in MT- (pink; fc>2) and in MT+ (blue; fc<-2). Grey dots represent features that are statistically insignificant (p- value>0.01), and/or have an insignificant fc. 7-Dehydrod. = 7-Dehydrodesmosterol; these are the only annotated features among those statistically significant.


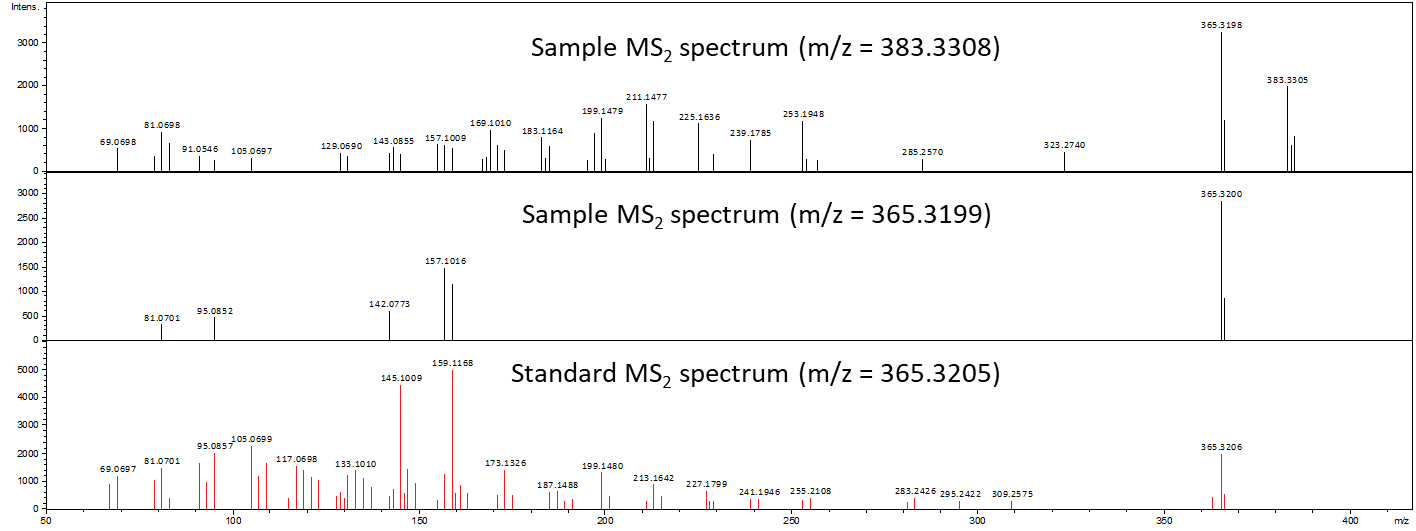


**Figure S4.** MS/MS spectra of 7-Dehydrodesmosterol in a sample (top and middle) and in the standard (bottom).
