# Supplementary figures and images for: A Metabolomics Exploration of the Sexual Phase in the Marine Diatom Pseudo-nitzschia multistriata
Source: Mar Drugs. 2020 Jun 14;18(6):313. doi: 10.3390/md18060313 (PMC7345340; doi:10.3390/md18060313)

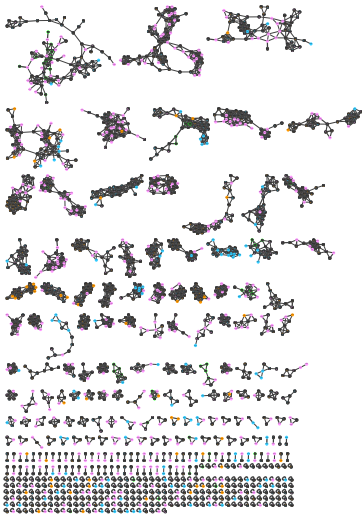

Supplement: Supplementary file 1 [file marinedrugs-18-00313-s001.zip › Figure 3a high res.pdf]
